# Developing a Bone-Mimicking Microenvironment: Surface Coating Method for Investigating Bone Remodeling *in Vitro*

**DOI:** 10.1021/acsbiomaterials.4c02330

**Published:** 2025-04-10

**Authors:** A. Sieberath, D. Eglin, C. M. Sprecher, A. M. Ferreira, P. Gentile, K. Dalgarno, E. Della Bella

**Affiliations:** a Newcastle University, Newcastle upon Tyne NE1 7RU, U.K.; b Mines Saint-Étienne, INSERM, U1059 Sainbiose, Saint-Étienne 42023, France; c AO Research Institute Davos, 7270 Davos Platz, Switzerland; d University Hospital Knappschaftskrankenhaus Bochum, 44892 Bochum, Germany

**Keywords:** osteoblast, osteoclast, coculture, bone remodeling, *in vitro*

## Abstract

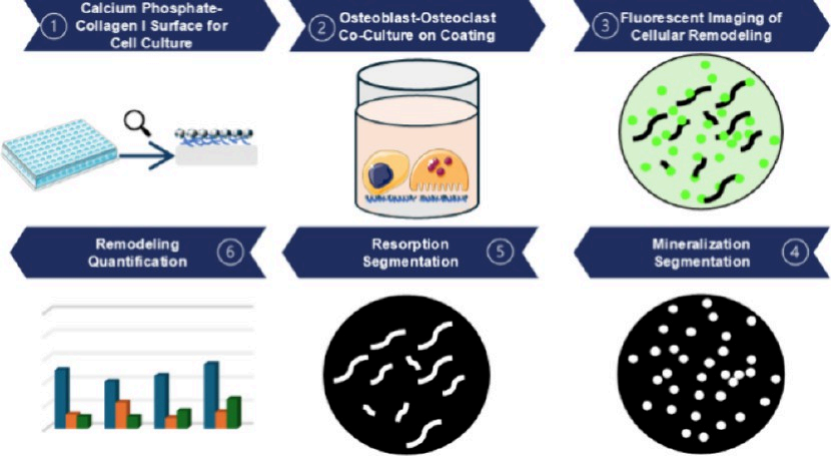

To investigate bone formation and resorption *in vitro*, it is essential to create bone-like microenvironments on cell culture substrates. Here, we present a coating technique to create such a microenvironment on cell culture plastic (CCP) multiwell plates for studying bone remodeling *in vitro*. Utilizing this coating, we have developed an assay to simultaneously measure cellular mineral formation and resorption in osteoblast and osteoclast coculture models. A composite matrix of collagen type I and carbonated apatitic calcium phosphate was deposited onto CCP in a reproducible manner using a 10× simulated body fluid solution (SBF) supplemented with type I collagen. qPCR analysis and cellular imaging using fluorescence microscopy demonstrated the promotion of osteogenic differentiation, cell attachment, and proliferation of human bone-marrow-derived mesenchymal stem cells on coated substrates. Moreover, human bone-marrow-derived mononuclear cells successfully differentiated into osteoclasts and resorbed the coated substrate. Using the developed coating, an osteoblast and osteoclast coculture system was established, enabling real-time monitoring of mineral formation and resorption. By providing a controlled and physiologically relevant *in vitro* model, this assay facilitates the screening of therapeutic compounds, the study of bone cell interactions, and the identification of factors influencing bone remodeling, thereby enhancing translational research in bone health.

## 1 Introduction

Bone undergoes a lifelong modeling and remodeling process. During bone modeling, the bone shape (*e*.*g*., trabecular orientation, thickening/thinning of the compact bone layer) is optimized according to the loading forces experienced on the bone, a homeostatic-like process in which bone strength and mineral content are maintained and balanced.^1^ This is achieved through the resorption of the old bone matrix by osteoclasts and the deposition of fresh mineralized bone matrix by osteoblasts. Osteoblasts and osteoclasts play vital complementary roles in bone modeling and remodeling, and work in coordination to maintain optimal bone quality.^2^

Osteoporosis occurs when there is a disruption in the coordinated activity of osteoblasts and osteoclasts during the bone remodeling process, leading to a state where bone resorption overcomes bone formation, resulting in a net decrease in bone matrix.^3^*In vitro*-based bone tissue systems and animal models have been used to explore the basic processes of healthy and pathological bone remodeling. Animal models of osteoporosis such as ovariectomized rodents and aged mice have been particularly valuable for preclinical drug testing, allowing researchers to investigate the effects of specific substances at the systemic level.^4−6^ However, the predictive value of preclinical osteoporosis studies remains limited due to species-specific differences in bone metabolism, as well as the high costs, low throughput, and ethical concerns associated with animal experiments.^7−10^

Therefore, it is crucial to create alternative models that accurately mimic *in vivo* cellular behavior and have the ability to predict human outcomes. This imperative aligns with the 3R principle (Replacement, Reduction, and Refinement), and is crucial for advancing new drug development and personalized treatment approaches.^11^

Given their pivotal role in bone formation and resorption, cocultures of osteoblasts and osteoclasts are essential for creating new *in vitro* models that could potentially yield more predictive results in preclinical drug testing than animal models of osteoporosis. Therefore, the development of *in vitro* models simulating the bone remodeling process may facilitate the development of novel therapeutic agents for the effective prevention and treatment of osteoporosis in the future.^12^ Several *in vitro* 2D osteoblast-osteoclast coculture models utilizing biomimetic substrates have been established as either direct or indirect cell contact models.^13−17^ While these systems provide valuable insights into the interactions among bone cell, they often lack the ability to simultaneously quantify mineral formation and resorption, limiting their usefulness for dynamic studies of bone remodeling. Current approaches for assessing osteoblast-mediated mineral formation in monoculture models rely on fluorescent calcium chelating dyes, such as Calcein Green^18−20^ or tetracycline hydrochloride.^21^ Osteoclast-mediated resorption is typically quantified by evaluating the degradation of mineral substrates, such as the Corning Osteo Assay Surface, using Von Kossa staining.^22^ However, these methods require separate assays for formation and resorption, making direct comparisons challenging and reducing throughput efficiency.

To address these limitations, we developed a coculture system that enables the quick and efficient evaluation of cellular mineral formation and resorption on a biomimetic cell culture substrate. Our approach involves coating cell culture plastic (CCP) using 10× simulated body fluid (SBF) and collagen to create a bone-like microenvironment. We evaluated the effects of this coating on the differentiation of bone marrow-derived mononuclear cells (BM-MNCs) into osteoclasts and bone marrow-derived human mesenchymal stem cells (BM-hMSCs) into osteoblasts. Compared to conventional hydroxyapatite coatings, our biomimetic coating is more effective in promoting cellular attachment, viability, proliferation, and osteogenic differentiation. Furthermore, our system allows for the simultaneous real-time tracking and quantification of both mineral formation and resorption using the fluorescent dye Calcein green, offering a novel and high-throughput approach for bone remodeling assays. This advancement has significant potential for drug screening applications, enabling more efficient preclinical evaluation of therapeutic agents targeting osteoporosis and other bone diseases.

## 2 Materials and Methods

### 2.1 10× SBF and 10× SBF Collagen Coating Process

10× SBF solution was prepared according to the method described by Tas and Bhaduri.^23^ The reagents described in Table 1 (all from Sigma-Aldrich; St. Louis, Missouri, United States; ACS quality) were added to 1900 mL of deionized water under constant stirring at room temperature (RT).

**Table 1:** Preparation of 2 L of 10× SBF Stock Solution

Order	Reagent	Amount (g)	Concentration (mM)
1	NaCl	116.886	1000
2	KCl	0.746	5
3	CaCl_2_ 2H_2_O	7.351	25
4	MgCl_2_ 6H_2_O	2.033	5
5	NaH_2_PO_4_	2.400	10

The solution was topped up with deionized water to a volume of 2 L. The coating was prepared by adding 30 mM of NaHCO_3_ to 50 mL of the stock solution to raise the pH of the solution to be in the range 6.35–6.0. After 5 min, the solution was added to CCP in multiwell plates and incubated at RT for 2 h. CCP samples were removed from the solution, washed with deionized water, and dried at room temperature. The coated surfaces were UV-sterilized for 30 min before cell culture experiments were started.

To generate the 10× SBF collagen coating, bovine type I collagen solution (6 mg/mL, Collagen Solutions, UK) was added to the 10× SBF stock solution at a concentration of 1 mg/mL before the addition of NaHCO_3_. Otherwise, the protocol remained unchanged. The pH rise during the coating procedure led to simultaneous formation and deposition of collagen fibers and calcium phosphate minerals.

### 2.2 Scanning Electron Microscopy (SEM) and Energy Dispersive X-ray Analysis (EDX)

All samples were mounted onto SEM specimen stubs and sputter-coated with a thin carbon layer. Surface coatings on CCP were cut from the plate using a saw. The working distance for imaging was set to 11.4 mm and the accelerating voltage ranged 3–8 kV. Images were captured in secondary electron mode with a Tescan Vega LMU SEM (Tescan). Energy-dispersive X-ray analysis (EDX, Oxford Instruments, Abingdon, UK) was used to assess both the coating composition and resorptive activity of osteoclasts.

### 2.3 X-ray Diffraction (XRD) Analysis

The deposited coating material was collected using a scraper and air-dried. The samples were then packed into a zero-background holder and loaded into a X’Pert diffractometer (PANalytical) equipped with an X’Celerator detector. A survey scan was performed to identify regions of interest in the diffraction spectra. Then, a high-resolution scan was performed in the determined region between 20 and 80 2θ with the following parameters: step size = 0.05°, time per step = 12 s, divergence slit = 1°, beam mask = 20 mm, and receiving slit = 0.5°. For phase analysis, the HighScorePlus software (Bruker, Karlsruhe, Germany) was used in combination with the crystal open database (COD).

### 2.4 Attenuated Total Reflection Fourier Transform Infrared Spectroscopy

The deposited coating was collected using a scraper and air-dried. The samples were then scanned in an attenuated total reflection Fourier-transform infrared spectrometer (ATR-FTIR; PerkinElmer, Waltham, MA, USA; Spektrum 2) in ATR mode at frequencies from 550 to 4000 cm^–1^ with 32 scans per spectrum. The nominal resolution was set to 4 cm^–1^. The anvil pressure applied to the ATR crystals was adjusted to 120 N/m^2^ for each sample. Each spectrum was collected in an air background. All spectra were obtained after baseline correction and in the wavenumber range of 550–4000 cm^–1^.

### 2.5 hMSCs Cell Culture

Y201 hTERT immortalized human mesenchymal stem cells (Y201 hMSCs) were used to assess cell viability on the coated substrates. Y201 hMSCs were cultured in growth medium consisting of DMEM (Thermo Fisher Scientific, Waltham, MA, USA) supplemented with 10% fetal bovine serum (FBS, Thermo Fisher Scientific) and 1% penicillin/streptomycin (P/S; Thermo Fisher Scientific). The culture medium was changed every two–three days and the cells were passaged at 80% confluency.

Primary bone marrow derived (BM)-hMSCs, isolated from 3 donors as previously described,^24^ were used to investigate the impact of 10× SBF collagen coating in comparison to a commercially available mineral cell culture substrate (Osteo Assay Surface, Corning Inc., Corning, NY, USA) on osteogenic differentiation. Bone marrow aspirates were obtained from patients who underwent spinal surgery at the Inselspital Bern (Bern Req-2016-00141). The Swiss Human Research Act does not apply to research involving anonymized biological material and/or anonymously collected or anonymized health-data.^25,26^ General Consent, which also covered anonymization of health-related data and biological material, was obtained from all cell donors.

BM-MSCs were seeded at a density of 1.0 × 10^4^ cells/cm^2^ and then cultured in 1 g/L glucose DMEM (Thermo Fisher Scientific) containing 10% MSC-qualified FBS (Corning) and 1% P/S (Thermo Fisher Scientific) for 7 days. The medium was changed to an osteogenic medium consisting of low-glucose DMEM with 10% FBS, 1% P/S, 50 μg/mL ascorbic acid 2-phosphate (Sigma-Aldrich), 5 mM β-glycerol phosphate (Sigma-Aldrich), and 10 nM dexamethasone-cyclodextrin complex (Sigma-Aldrich) to induce osteogenic differentiation. Cells maintained in low-glucose medium containing 10% FBS and 1% P/S were used as controls. Differentiation was followed for 21 days, and the medium was changed every second day.

### 2.6 SaOS-2 Cell Culture

The human osteoblast tumor cell line SaOS-2 was cultured according to the following conditions. Cells were seeded at a density of 1.0 × 10^4^ cells/cm^2^ in T-75 cell culture flasks in McCoy’s 5a cell culture medium supplemented with 2 mM glutamine, 10% FBS and 1% P/S. Cells were passaged when they reached 80% confluency. For the experiments described in section 6.2, SaOS-2 cells were seeded at 1.5 × 10^4^ cells/cm^2^ and cultured for 4 weeks in osteogenic medium consisting of McCoy’s 5a cell culture medium, 10% FBS, 1% P/S, 50 μg/mL ascorbic acid 2-phosphate, 5 mM β-glycerol phosphate and 10 nM dexamethasone.

### 2.7 Osteoclast Cell Culture

The 10× SBF collagen coating was benchmarked against the current state-of-the-art hydroxyapatite-coated cell culture surface (Corning Osteo Assay Surface). Bone marrow-derived mononuclear cells (BM-MNC) were used as the source of osteoclast precursor cells. Mononuclear cells obtained after density gradient centrifugation were seeded at a density of 2.5 × 10^5^ cells/cm^2^ and incubated overnight in αMEM (Thermo Fisher Scientific) supplemented with 10% FBS and 1% P/S. The next day, nonadherent cells were collected and seeded at 2.5 × 10^5^ cells/cm^2^ in αMEM supplemented with 10% FBS, 1% P/S, and 30 ng/mL M-CSF (Peprotech, Hamburg, Germany). After 3 days osteoclast precursors adhered to the flask, and the medium was changed. The osteoclast precursor cells were then expanded in the same medium until day 7 with the medium refreshed every two–three days. The medium was then changed to α-MEM supplemented with 10% FBS, 1% P/S, 30 ng/mL M-CSF, and 60 ng/mL RANKL (Peprotech) to induce osteoclast formation. Osteoclasts formed after four–seven days of RANKL stimulation. At 7 and 14 days, the gene expression levels of osteoclastic markers and Tartrate-resistant acid phosphatase (TRAP) secretion were analyzed, while TRAP staining and coating resorption capability were assessed at 14 days.

Alizarin Red and Calcein Green staining were compared to evaluate new mineral deposition by osteoblast-like cells on 10× SBF collagen-coated substrates. For this purpose, SaOS-2 cells were seeded at 1.5 × 10^4^ cells/cm^2^ and cultured for 4 weeks in osteogenic medium consisting of McCoy’s 5a cell culture medium, 10% FBS, 1% P/S, 50 μg/mL ascorbic acid 2-phosphate, 5 mM β-glycerol phosphate, and 10 nM dexamethasone.

### 2.8 Live/Dead Staining and Cell Morphology Analysis

Live/Dead staining (Thermo Fisher Scientific) was used to cell viability on mineralized surfaces. Staining was performed according to the manufacturer’s protocol. Briefly, Y201 hTERT were seeded at a density of 5 × 10^3^ cells/cm^2^, and the procedure was performed after 24 h. After a washing step, cells were stained using a working solution containing the dyes Calcein-AM (2 μM) and ethidium homodimer-1 (4 μM) in PBS. The cells were incubated in the staining solution for 20 min at 37 °C and then viewed under a fluorescence microscope (EVOS Microscope M5000 Imaging System, Thermo Fisher Scientific).

Pictures were captured to evaluate cell spreading and cell circularity on the surfaces of interest. A circular and condensed cell shape indicated poor cellular attachment, whereas a spread-out and elongated shape indicated strong cell attachment. Images were analyzed using ImageJ, according to the following protocol. First, images were converted into 8-bit grayscale images and then binarized using an appropriate threshold to distinguish between the cells and background. Using the “Analyse Particles” function in ImageJ, the covered area and circularity of each cell was measured. Circularity is a relative measure that indicates whether cells elongate or remain circular on a substrate. A value close to 1 indicates a round cell shape, whereas a value close to 0 indicates an elongated cell shape. The circularity is defined as follows (eq 1):
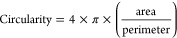
1The area covered by the cells was chosen as an absolute measurement of the extent of cell spreading. Only single cells were analyzed, and cells on the edges of the images were excluded. At least three images per surface were analyzed, resulting in at least 60 cells per surface of interest.

### 2.9 Lactate Dehydrogenase (LDH) Cytotoxicity Assay

The lactate dehydrogenase (LDH) cytotoxicity assay (Sigma-Aldrich) was used to evaluate the potential cytotoxicity of the 10× SBF- and 10× SBF collagen coatings. The assay was performed according to the manufacturer’s instructions. Y201 hMSCs were seeded on the coated substrates and CCP at a density of 1.5 × 10^4^ cells/cm^2^. The cells were incubated overnight, and the assay was performed the following day. Lysis buffer (supplied by the assay supplier) was added to the wells serving as the maximum LDH control and incubated for 45 min at 37 °C. Fifty microliters of each sample (medium control, CCP control, maximum LDH control, and coated substrates) were added to a clear 96-well flat-bottom plate in triplicate wells. Fifty microliters of the LDH assay reaction mixture was added to each well and mixed by gentle tapping. After 30 min of incubation at room temperature in the dark, 50 μL of stop solution was added to each sample. Absorbance at 490 and 680 nm was measured using a spectrophotometer (CLARIOstar, BMG Labtech, Germany). To determine LDH activity, the absorbance at 680 nm (background signal) was subtracted from the absorbance at 490 nm. The percentage of cytotoxicity was calculated using the following formula (eq 2):
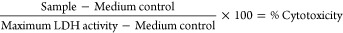
2All samples were analyzed in triplicate.

### 2.10 Metabolic Activity

The CellTiter-Blue Cell Viability Assay (Promega, Madison, WI, USA) was used to estimate metabolic activity of cells on coated and noncoated CCP. Y201 hMSCs were seeded at a density of 5 × 10^3^ cells/cm^2^ and analyzed after one, three and 7 days in triplicates at each time point. The cells were incubated in CellTiter-Blue solution according to the instructions of the manufacturer for four hours at 37 °C. Fluorescence at 530/590 nm (excitation/emission) was measured using a microplate reader (CLARIOstar, BMG Labtech, Germany), and each sample was analyzed in triplicate.

### 2.11 Osteoblast–Osteoclast Coculture

Osteoblast-osteoclast coculture models (using differentiated BM-hMSCs as a preosteoblast source) were established on 10× SBF collagen-coated cell culture dishes using three different osteoblast-to-osteoclast ratios:

1. Osteoblast monoculture.
2. osteoblasts (OB): osteoclast (OC) 200:1
3. OB:OC 7:1

The OB:OC 200:1 ratio was chosen according to the reported composition of the basic multicellular unit during osteon remodeling.^27,28^ The 7:1 ratio was selected according to a previously reported osteoblast-osteoclast ratio in patients suffering from postmenopausal osteoporosis.^29^

BM-hMSCs were differentiated on CCP under osteogenic conditions as described above. After 3 weeks, the cells were detached using a 0.25% trypsin solution (Thermo Fisher Scientific). Once the upper cell layer was detached, trypsin activity was stopped by adding growth medium, and the cell suspension was collected and centrifuged at 200 × g. The supernatant was aspirated, and the cells were resuspended in prewarmed osteogenic medium. Predifferentiated BM-hMSCs were seeded onto 10× SBF collagen-coated plates at a density of 2 × 10^4^ cell/cm^2^. The cells were maintained for further 3 weeks under osteogenic conditions to allow mineral deposition on the substrate. Predifferentiated osteoclasts (cultured for 7 days with M-CSF, followed by 6 days of M-CSF+RANKL stimulation as described above) were added to differentiated BM-hMSCs. The coculture was maintained for further 7 days in α-MEM supplemented with 10% FBS, 1% P/S, 30 ng/mL M-CSF, 60 ng/mL RANKL, 50 μg/mL ascorbic acid 2-phosphate, 5 mM β-glycerol phosphate, and 10 nM dexamethasone.

### 2.12 Tartrate-Resistant Acid Phosphatase Staining

TRAP staining and quantification were carried out using the leukocyte TRAP kit (Sigma-Aldrich) according to the manufacturer’s instructions. Briefly, osteoclasts were fixed by immersion in fixative solution (25 mL Citrate Solution, 65 mL acetone, and 8 mL of 37% (v/v) formaldehyde) for 30 s and then rinsed thoroughly in deionized water. The cells were then incubated for 1 h at 37 °C in TRAP staining solution. The staining solution was then removed, and the cells were rinsed thoroughly with deionized water. The cell nuclei were counterstained with HOECHST 33258.

TRAP secreted into the cell culture supernatant was used as a quantitative measure of osteoclast formation. The TRAP staining solution (80 μL) was added to the cell culture supernatant (20 μL). The solution was then incubated for 1 h at 37 °C. The reaction was stopped using 100 μL of a 0.3 M NaOH solution. The absorbance was measured at 540 nm using a microplate reader (CLARIOstar).

### 2.13 Alkaline Phosphatase Activity Assay

Cells were washed with PBS and lysed with lysis buffer (0.1% Triton X100 in 10 mM Tris-HCl, pH 7.4) for at least 60 min at 4 °C on an orbital shaker. Lysates were mixed with alkaline phosphatase (ALP) substrate solution, vortexed, and immediately incubated at 37 °C in the dark in a heating block for 15 min. The reaction was stopped by adding a 0.1 M NaOH solution. Aliquots were transferred from each sample in duplicate to a 96-well plate and the absorbance at 405 nm was recorded. ALP concentration in each sample was determined using a standard curve. Total ALP enzyme activity per minute was calculated for each sample. ALP activity was normalized to the DNA content of each sample. The CyQuant DNA quantification assay (Thermo Fisher Scientific) was performed according to the manufacturer’s instructions.

### 2.14 Real-Time Polymerase Chain Reaction (qPCR)

Standard TRI reagent extraction with 1-bromo-3-chloropropane (Sigma-Aldrich) was used to isolate total RNA. After phase separation, RNA was precipitated from the aqueous phase by adding 2-propanol (Sigma-Aldrich). RNA pellets were washed with 75% EtOH and reconstituted in diethylpyrocarbonate (DEPC)-treated H_2_O. Total RNA concentration and purity were evaluated using a NanoDrop One UV spectrophotometer (Thermo Fisher Scientific). For total gene expression, TaqMan Reverse Transcription reagents (Applied Biosystems, Foster City, CA, USA) were used to synthesize cDNA from 1000 ng of total RNA. Osteoblast and osteoclast differentiation markers were analyzed by qPCR. TaqMan Gene Expression Master Mix (Applied Biosystems) in a QuantStudio 7 Flex Real-Time PCR system (Applied Biosystems) was used to amplify target genes by applying the following protocol: 2 min at 50 °C; 10 min at 95 °C; 40 cycles of 15 s at 95 °C and 1 min at 60 °C. TaqMan gene expression assays (Thermo Fisher) or custom-designed primers and probes (Microsynth AG, Balgach, Switzerland) were used to test the genes of interest (Table 2). The results were expressed as 2^–ΔCt^, with *RPLP0* used as the reference gene.

**Table 2:** TaqMan Gene Expression Assays and/or Custom-Designed Primers and Probes Information

Gene ID	Forward sequence (or assay ID^a^)	Reverse sequence	Probe sequence^b^
*ACP5*	Hs00356261_m1		
*BGLAP*	5′-AAG AGA CCC AGG CGC TAC CT-3′	5′-AAC TCG TCA CAG TCC GGA TTG-3′	5′-ATG GCT GGG AGC CCC AGT CCC-3′
*CTSK*	Hs00166156_m1		
*MMP9*	Hs00957562_m1		
*IBSP*	Hs00173720_m1		
*RPLP0*^c^	5′-TGG GCA AGA ACA CCA TGA TG-3′	5′-CGG ATA TGA GGC AGC AGT TTC-3′	5′-AGG GCA CCT GGA AAA CAA CCC AGC-3′
*RUNX2*	5′-AGC AAG GTT CAA CGA TCT GAG AT-3′	5′-TTT GTG AAG ACG GTT ATG GTC AA-3′	5′-TGA AAC TCT TGC CTC GTC CAC TCC G-3′
*SOX9*	Hs00165814_m1		
*SP7*	5′-CCT GCT TGA GGA GGA AGT TCA-3′	5′-GGC TAG AGC CAC CAA ATT TGC-3′	5′-TCC CCT GGC CAT GCT GAC GG-3′
*TNFRSF11*	Hs00243522_m1		

a TaqMan Gene expression assay (Thermo Fisher Scientific). 5′ modification: FAM. 3′ modification: NFQ-MGB.

b 5′ modification: FAM. 3′ modification: TAMRA.

c Reference gene.

### 2.15 Calcein Staining of Minerals and Remodeling Quantification

Calcein Green powder (Sigma-Aldrich) was dissolved in 100 mM KOH to prepare a 30 mM stock solution. The stock solution was further diluted with water to generate a 6 mM working solution. The working solution was sterile filtered using a 0.2 μm-pore filter. The Calcein Green working solution was added directly to the cell culture medium at a final concentration of 6 μM. The Calcein Green-supplemented cell culture medium was refreshed every two–three days. To further reduce the fluorescent background and increase the imaging quality, the samples were washed twice in calcein-free medium and then kept in calcein-free medium to capture images (EVOS2 Cell Imaging System, Thermo Fisher Scientific). For the time-lapse imaging, Calcein Green prestained mineral surfaces seeded with OCs were kept in a microscope on-stage incubator under a controlled atmosphere (37 °C, 5% CO_2_; EVOS Onstage Incubator, Thermo Fisher Scientific). Images were acquired every 20 min for 22 h.

Image analysis of Calcein Green-stained cell culture mineral surfaces was used to monitor cell remodeling activity in OC monocultures and OB-OC cocultures with the following protocol. Images were loaded into ImageJ (NIH, Bethesda, MD, USA) and thresholded to segment the bright mineral deposits from the background fluorescence of the 10× SBF collagen coating. The segmented mineral deposits were measured using the “Analyse Particle” function in ImageJ. To reduce visual noise, the minimum particle size for the analysis was set to 50 μm^2^. A similar process was used to analyze the cellular resorption sites. A low threshold was applied to segment the resorption sites from the remaining coating. Measurement of the segmented resorption sites was carried out using the “Analyse Particles” function in ImageJ. To reduce the visual noise in the analysis, the minimum particle size for resorption site analysis was set to 100 μm^2^. The parameters assessed for formation and resorption sites were “Average size remodeling sites”, “Number of remodeling sites”, and “Total area of remodeling sites”.

### 2.16 Alizarin Red Staining

Alizarin Red staining was used as a comparative method to the fluorescent calcein staining for the detection of cellular mineralization and resorption on the 10× SBF collagen-coated CCP. The samples were washed three times for 5 min with H_2_O. Afterward, 0.2 mL of a 40 mM Alizarin Red solution (Sigma-Aldrich) at pH 6.4 was added to each well and incubated for 1 h at RT on a rotating plate. Subsequently, the samples were washed at least five times with H_2_O until all dye residues were removed. The samples were then covered with H_2_O and inspected using an inverted light microscope (EVOS2 Cell Imaging System, Thermo Fisher Scientific).

### 2.17 Statistical Analysis

Statistical analysis was performed using GraphPad Prism version 10.0.2 (GraphPad Software LLC, Boston, MA, USA). All measurements are reported as mean ± standard deviation. Experiments consisting of two groups were analyzed using an unaired *t* test and experiments with three or more groups were analyzed using the one-way ANOVA test. Multiple comparison Bonferroni posthoc tests were performed to determine which groups were significantly different from one another. Results with *p* values <0.05 were considered as statistically significant.

## 3 Results

### 3.1 Development of Apatitic Calcium Phosphate Collagen Composite Surface Coating

We aimed to develop a calcium phosphate coating that promotes cell attachment and provides a bone-like environment to cells, thus allowing for long-term culture and osteogenic differentiation of BM-hMSCs.

**Figure 1:**
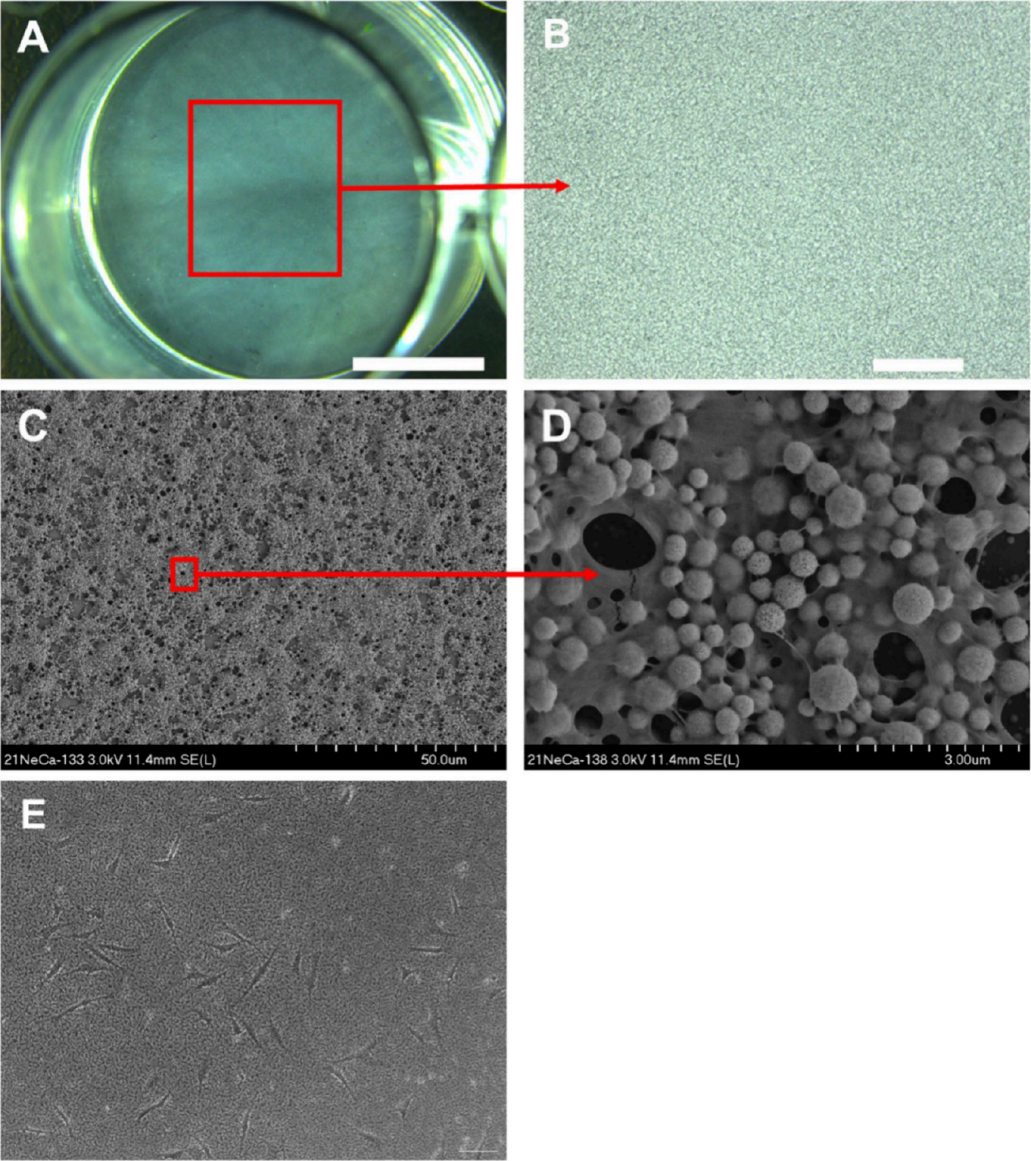
10× SBF collagen coating morphology. (A) Overview of the bright-field image of a coated well of a 24-well plate (scale bar 5 mm). (B) Higher-magnification bright-field image of the coated well (scale bar 200 μm). (C) Low-magnification SEM image of a coated well (1000×, scale bar 50 μm). (D) High-magnification SEM image a coated well (15000×, scale bar 3 μm). (E) Phase contrast image of the coating seeded with Y201 hMSCs (scale bar 100 μm).

The macroscopic morphology of 10× SBF collagen-coated CCP is presented in Figure 1A,B, which shows the formation of a thin matrix on the CCP surface after the coating process.

SEM images of the coated polymer surface in Figure 1C,D show that the developed coating process resulted in the deposition of a dense calcium phosphate collagen type I composite matrix (approximately 1 μm thick, see Supplementary Data Figure S2). Figure 1E shows Y201 hMSCs seeded on a coated CCP substrate to evaluate cell visibility using a conventional inverted phase-contrast microscope during cell culture, demonstrating that the coated CCP substrates allow the visualization of cell morphology during cell culture.

**Figure 2:**
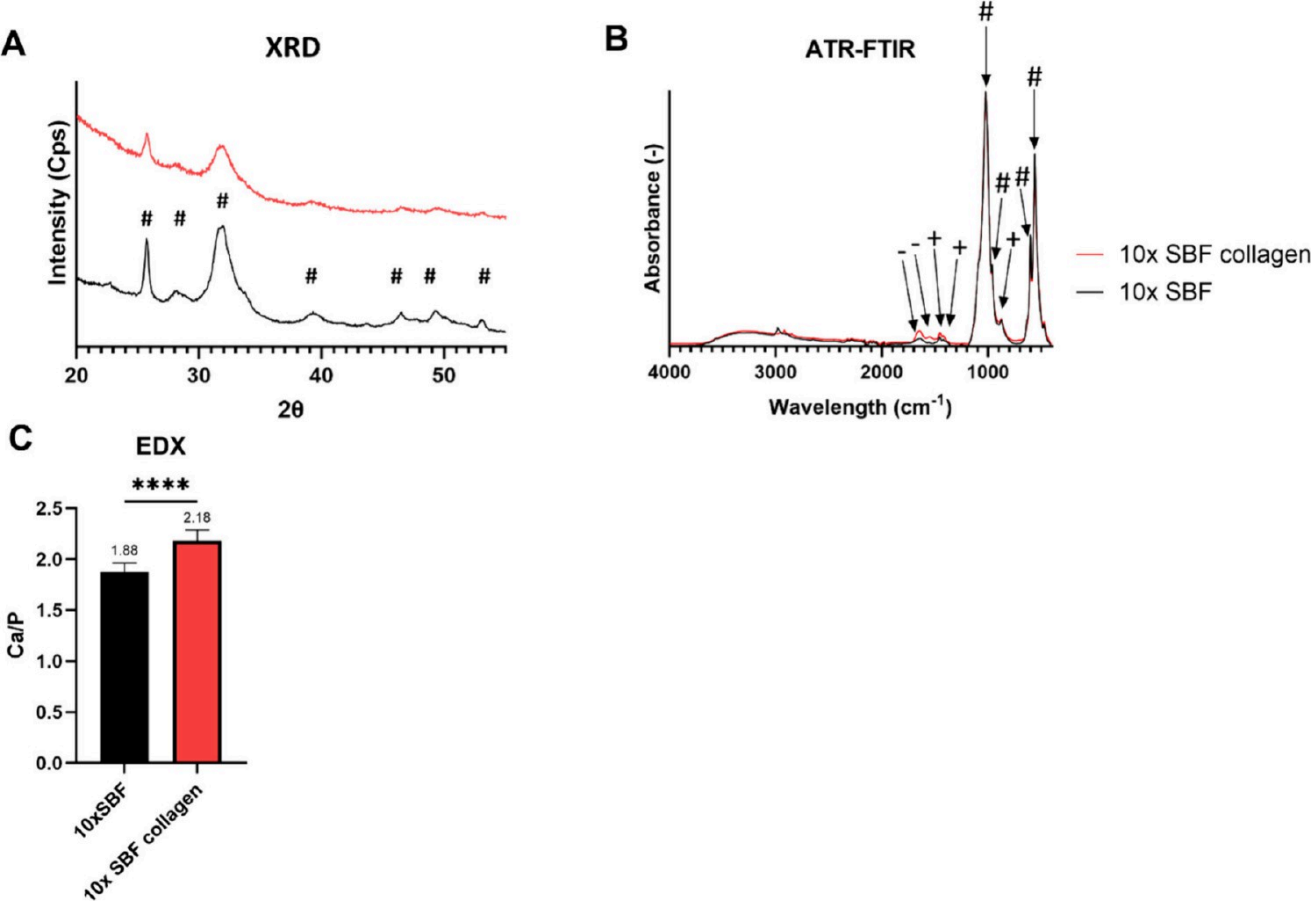
Calcium phosphate phase identification of 10× SBF collagen and 10× SBF by XRD, ATR-FTIR, and EDX analyses. (A) XRD analysis; # indicates characteristic hydroxyapatite peaks (COD reference 96-230-0274). (B) Recorded ATR-FTIR spectra. (C) Ca/P ratio of 10× SBF and 10× SBF collagen coating as calculated by EDX analysis. Statistical test: unpaired *t* test. 10× SBF: *n* = 9, 10× SBF collagen *n* = 12. ****: *p* < 0.0001.

Calcium phosphate phase identification and characterization of the 10× SBF collagen coating were carried out using X-ray diffraction (XRD), ATR-FTIR, and EDX analyses (Figure 2) and compared to the 10× SBF coating without collagen.

The XRD pattern and subsequent phase identification suggested that the 10× SBF collagen coating contained apatitic calcium phosphate (hydroxyapatite reference peaks (#), COD reference 96-230-0274, Figure 2A). The broad nature of the recorded peaks indicates the low crystallinity and amorphous state of the deposited apatitic calcium phosphate. The ATR-FTIR spectra of the coating revealed peaks at 560, 600, 960, and 1020 cm^–1^ (#) corresponding to PO_4_ and peaks at 875 and 1420/1440 cm^–1^ (+) corresponding to CO_3_ (Figure 2B). The CO_3_ peaks suggest the presence of carbonate-substituted apatitic calcium phosphate. The bands at 1550 and 1650 cm^–1^ (−) can be attributed to amide 1 and 2 connections, respectively, indicating the presence of collagen in the coating. Quantitative analysis using EDX indicated a Ca/P ratio of 2.18 in the deposited minerals of the 10× SBF collagen coating (Figure 2C).

XRD analysis of the 10× SBF coating (devoid of collagen) revealed the deposition of more crystalline apatitic calcium phosphate particles, as indicated by the sharper and well-defined diffraction peaks compared to the 10× SBF collagen coating. The recorded ATR-FTIR spectrum of the 10× SBF coating shows a similar pattern to that of the 10× SBF collagen coating but lacks the amide 1 and 2 bands at 1550 and 1650 cm^–1^. The quantified Ca/P ratio of the 10× SBF coating was 1.88, which was lower than the recorded ratio of the 10× SBF collagen coating.

### 3.2 10× SBF Collagen Coating Promotes Attachment and Proliferation of hMSCs

**Figure 3:**
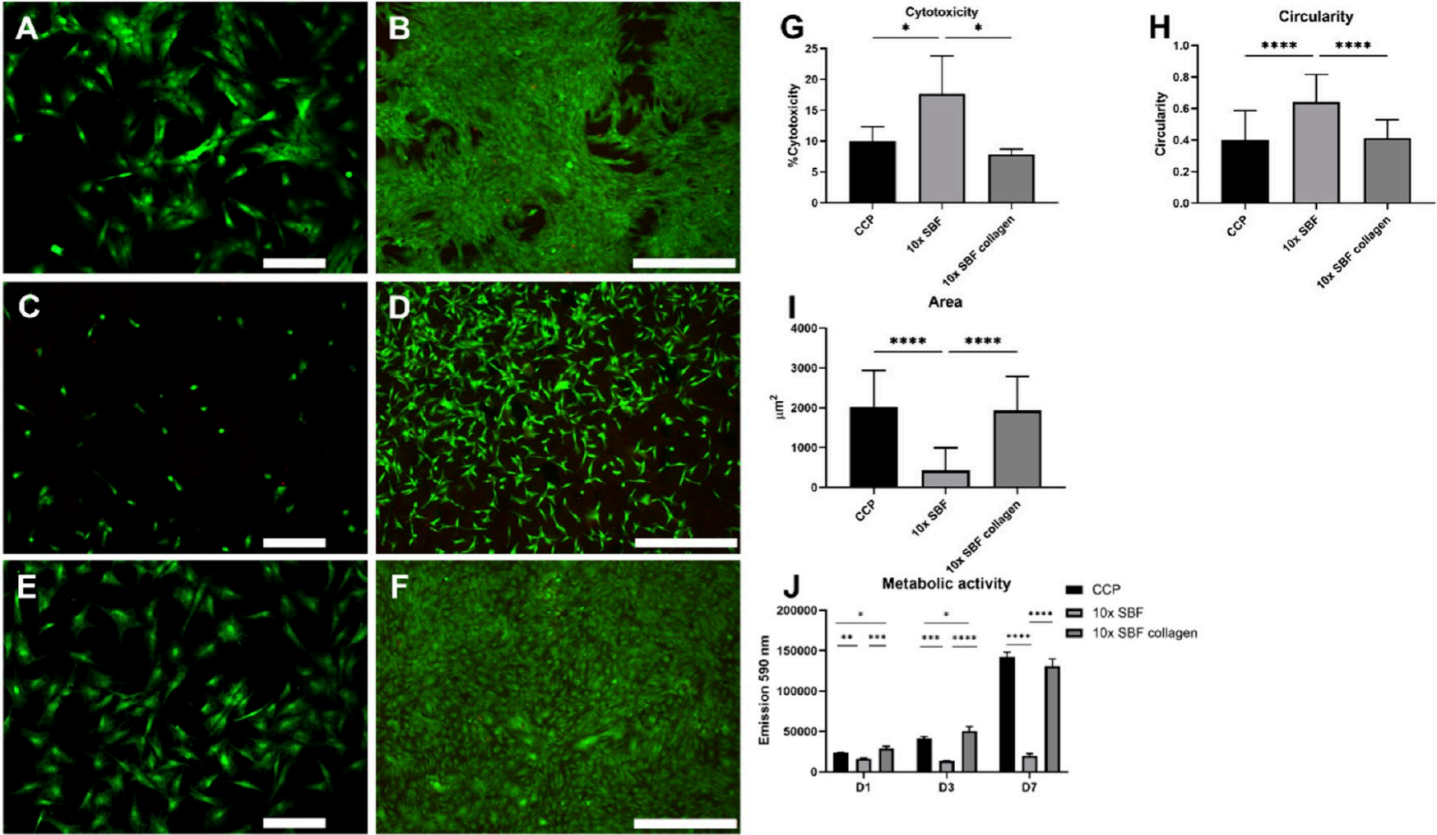
Y201 hMSCs viability on different growth substrates. (A, B) Live/Dead staining of cells cultured on CCP for 1 day (A) and 7 days (B). (C, D) Live/Dead staining of cells cultured on 10× SBF coated CCP after 1 day (C) and 7 days (D). (E, F) Live/Dead staining of cells cultured on 10× SBF collagen-coated CCP after 1 day (E) and 7 days (F). (G) LDH toxicity assay after 1 day. (H) Mean cell circularity and (I) mean cell surface area. (J) Metabolic activity measurements on days 1 and 3 and 7 days after seeding. Scale bars = 200 μm (A, C, E) and 500 μm (B, D, F). Statistical test: one way ANOVA with multiple comparison Bonferroni post-hoc test. Cytotoxicity: CCP, *n* = 5; 10× SBF, *n* = 8; 10× SBF collagen *n* = 3. Circularity and area: CCP, *n* = 122; 10× SBF, *n* = 80; 10× SBF collagen *n* = 153. Metabolic activity: *n* = 3. Statistical significance: *p < 0.05, **p < 0.01, ***p < 0.001, ****p < 0.0001.

The viability and proliferation of cells cultured on the 10× SBF collagen coating were compared with those cultured on CCP and 10× SBF coating. Live/Dead staining at day one post seeding showed a high cell viability and a low cell density on all three surfaces (Figure 3A,C,E). Comparably more dead cells were detected on the 10× SBF coating, which was further confirmed by the LDH cytotoxicity assessment (Figure 3G). Seeding cells on the 10× SBF coating caused higher cytotoxicity (18%) than the 10× SBF collagen coating (8%) and the CCP control (12%), with no significant different between the CCP and the 10× SBF collagen coating.

Cell morphology was analyzed as a parameter for the quality of cell attachment 1 day after seeding. Cells cultured on the 10× SBF coated surface (Figure 3C) had a rounder morphology in comparison to cells on the CCP control (Figure 3A), indicating poor cell attachment. Cells seeded on the collagen supplemented 10× SBF mineral surface (Figure 3E) showed an elongated morphology similar to that of the CCP control, which was indicated by a similar circularity index (Figure 3 H). The surface area of cells on the 10× SBF coated surface was significantly smaller than on the 10× SBF collagen and CCP surfaces (Figure 3I). However, there was no significant difference between cells cultured on 10× SBF collagen and CCP (Figure 3 I).

Live/Dead staining after 7 days of culture indicated high viability and an increase in cell number on each surface type (Figure 3B,D,F). Cells on CCP grew into a confluent monolayer (Figure 3B), while cells on the 10× SBF coating were less abundant and showed more red fluorescence signals, indicating dead cells (Figure 3D). Cells cultured on the 10× SBF collagen surface were abundant and grew into a confluent monolayer, similar to the results observed on CCP (Figure 3F). Also, fewer dead cells were detected than in the 10× SBF group.

Measurement of metabolic activity (Figure 3J) showed that cells seeded on the 10× SBF collagen surface had a total activity comparable to that of the CCP control, indicating a similar number of viable cells. Moreover, the activity in both groups increased between D1 and D7, suggesting an increase in the cell number over the culture period. In contrast, the cells seeded on the 10× SBF coated surface had a relatively lower total metabolic activity on D1, D3, and D7, and the activity increased only slightly during the 7 days of culture.

Taken together, these results show that 10× SBF collagen coating promotes cell attachment and proliferation and thus represents a superior growth substrate compared to a plain calcium phosphate-coated surface (10× SBF).

### 3.3 10× SBF Collagen Coating Supports Osteoclastic Differentiation of BM-Monocytes

**Figure 4:**
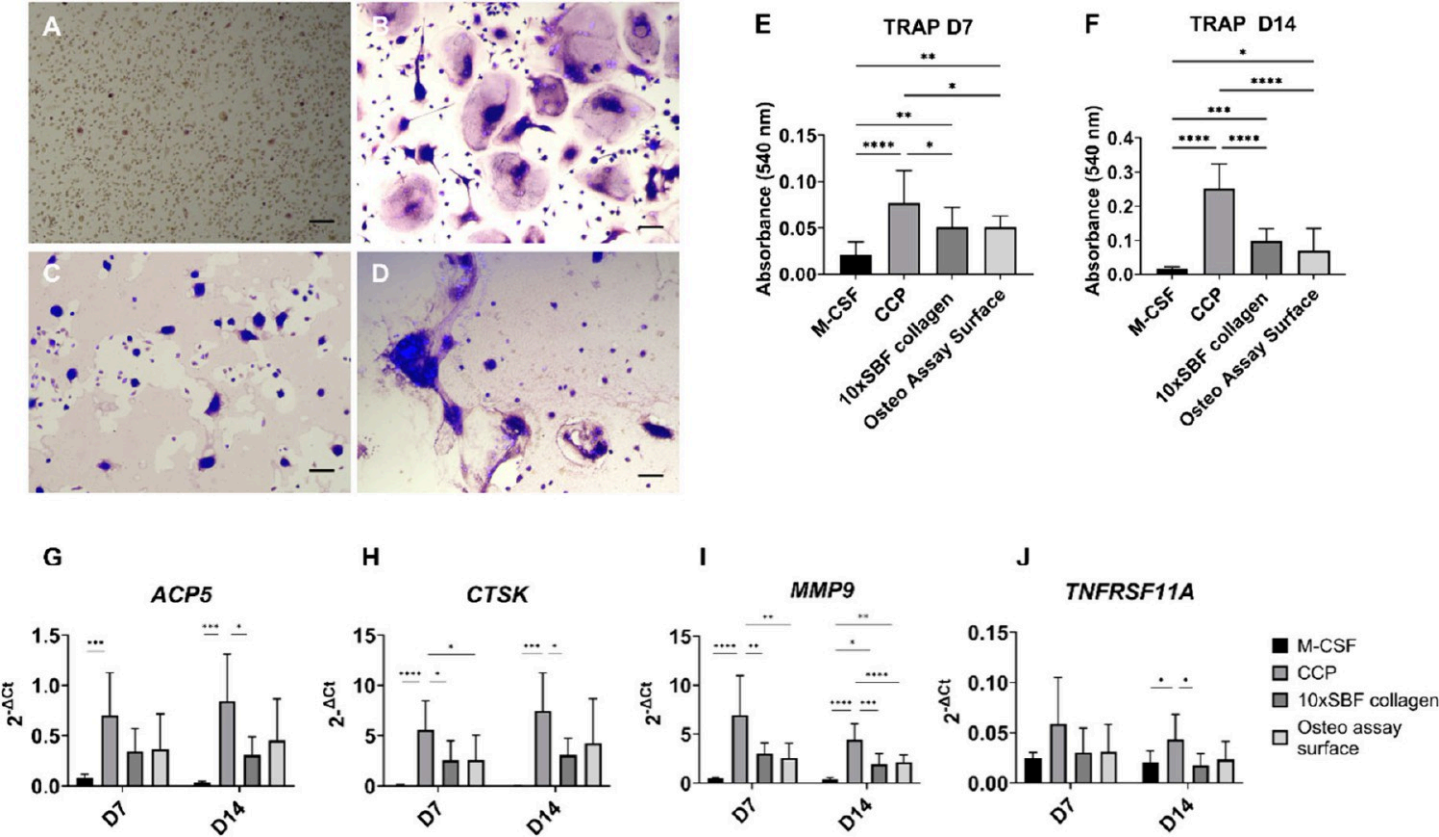
Osteoclast differentiation of BM-MNCs (*n* = 3 donors) on mineral surfaces after 14 days of M-CSF and RANKL stimulation. (A) TRAP staining of M-CSF only stimulated osteoclast precursors on CCP. (B) TRAP staining of M-CSF- and RANKL-stimulated osteoclast precursors on CCP. (C) TRAP staining of M-CSF- and RANKL-stimulated osteoclast precursors on the Osteo Assay Surface. (D) TRAP staining of M-CSF- and RANKL-stimulated osteoclast precursors on 10× SBF collagen coating. Scale bar = 100 μm. (E) Supernatant TRAP quantification after 7 days of culture of BM-MNCs derived osteoclasts. (F) Supernatant TRAP quantification after 14 days of culture. (G–J) Gene expression levels of osteoclast marker genes after 7 and 14 days of differentiation on mineral surfaces. (G) *ACP5*, (H) *CTSK*, (I) *MMP9*, and (J) *TNFRSF11A*. Statistical test: one way ANOVA with multiple comparison Bonferroni posthoc test. Statistical significance: *p < 0.05, **p < 0.01, ***p < 0.001, ****p < 0.0001.

The 10× SBF collagen was evaluated for its effect on differentiation of osteoclasts from BM-MNC and compared to the Corning Osteo Assay Surface (Figure 4). BM-MNCs stimulated with only M-CSF (undifferentiated control, Figure 4A) did not exhibit a TRAP-positive staining, while RANKL-stimulated cells on CCP, Corning Osteo Assay Surface and 10× SBF collagen were TRAP-positive (Figure 4B–D). Furthermore, resorption sites were visible on the 10× SBF collagen coating and Osteo Assay Surface, indicating the resorbability of both substrates and their potential to assess cell functionality (Figure 4C,D). Osteoclasts differentiated on CCP showed a higher TRAP release after 7 and 14 days, suggesting a higher number or activity of osteoclasts in comparison to cells differentiated on 10× SBF collagen and Osteo Assay Surface (Figure 4E,F). The results of the gene expression analysis of the osteoclast-specific marker genes *ACP5*, *CTSK*, *MMP9*, and *TNFRSF11A* (Figure 4G–J) showed that the expression of these four genes was highest on the CCP surface in comparison to both mineral surfaces after 7 and 14 days of culture. This observation is in line with the TRAP staining and quantification results. Osteoclasts on both mineral surfaces showed lower but similar levels of expression of the investigated genes at both time points.

**Figure 5:**
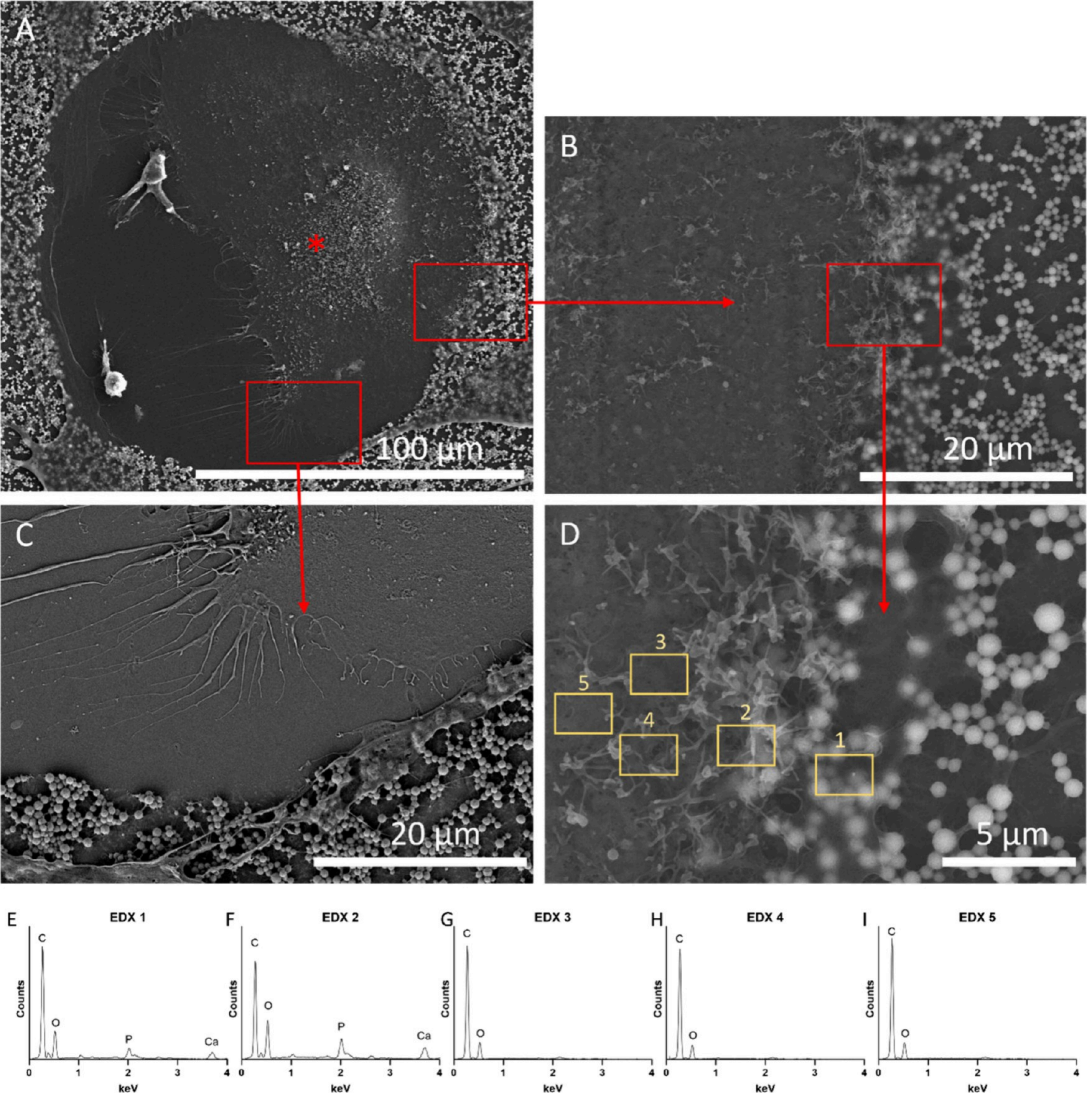
SEM and EDX analyses of an osteoclast on 10× SBF collagen-coated CCP, *n* = 2. (A) Overview of an osteoclast in a resorption site (scale bar 100 μm). (*) Indicates the formation of the secretory domain. (B) High-magnification image of the osteoclast side facing the direction of movement (scale bar 20 μm). (C) High-magnification image of the trailing side of the osteoclast (scale bar 20 μm). (D) EDX analysis of the interaction between the osteoclast and the mineral substrate (scale bar 5 μm). (E–I) EDX spectra acquired from Regions of Interest (ROIs) 1–5.

SEM imaging was used to further analyze osteoclast morphology and interaction with the 10× SBF collagen mineral substrate (Figure 5). Figure 5A shows an overview of an osteoclast in a resorption site on the mineral surface. Figure 5B shows a high-magnification image of the leading osteoclast side which faces toward the cell moving direction. On this side, the cell showed visible villi-like structures on the basal membrane, especially where the edges of the cell body started to cover the mineral surface. The concentration of villi-like structures (*) in the center of the osteoclast cell body indicates the formation of the secretory membrane domain (Figure 5A). At the trailing cell end (Figure 5C) retraction fibers and the resorption site are visible. Mineral resorption of the osteoclast was analyzed with EDX at the leading cell edge at locations 1–5 shown in (Figure 5D). The EDX spectra 1–2 (Figure 5G–I) at the cell front showed signals for calcium and phosphorus while spectra 3–5, which were located toward the cell center, did not exhibit signals for calcium and phosphorus.

### 3.4 10× SBF Collagen Coating Promotes Osteogenic Differentiation in BM-hMSCs

**Figure 6:**
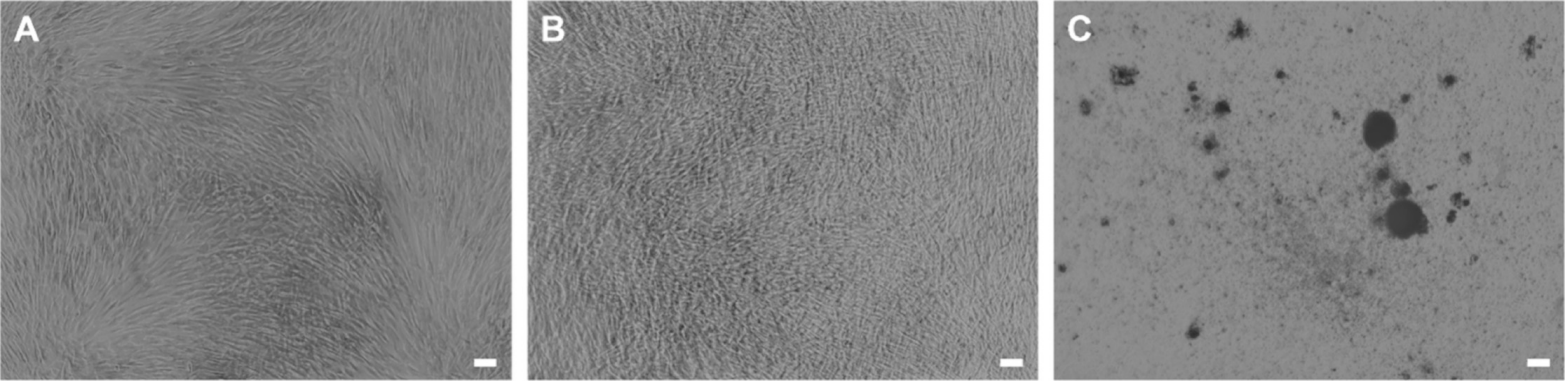
Phase contrast images of BM-hMSCs after 21 days of osteogenic differentiation on different substrates, *n* = 3. (A) Undifferentiated control on CCP. (B) Osteogenic control on CCP. (C) MSCs cultured under osteogenic conditions on the Osteo Assay Surface. Scale bar 100 μm.

We studied the Corning Osteo Assay Surface’s effect on BM-hMSCs osteogenic differentiation. Figure 6 compares the cell morphology after 21 days of culture on different substrates. The undifferentiated control (Figure 6A) and osteogenic control on CCP (Figure 6B) both showed cell growth and formed confluent monolayers. When seeded on the Osteo Assay Surface, notable changes were seen. After 1 week, cells detached and formed spherical clusters. By day 21, most of the cells had detached, leaving behind debris in the medium (Figure 6C).

For this reason, the Osteo Assay Surface was deemed unsuitable as a substrate for promoting osteogenic differentiation of BM-hMSCs. The detachment and formation of cellular agglomerates suggests a lack of attachment sites between the cells and the Osteo Assay Surface, leading to an unfavorable environment for osteogenic differentiation.

**Figure 7:**
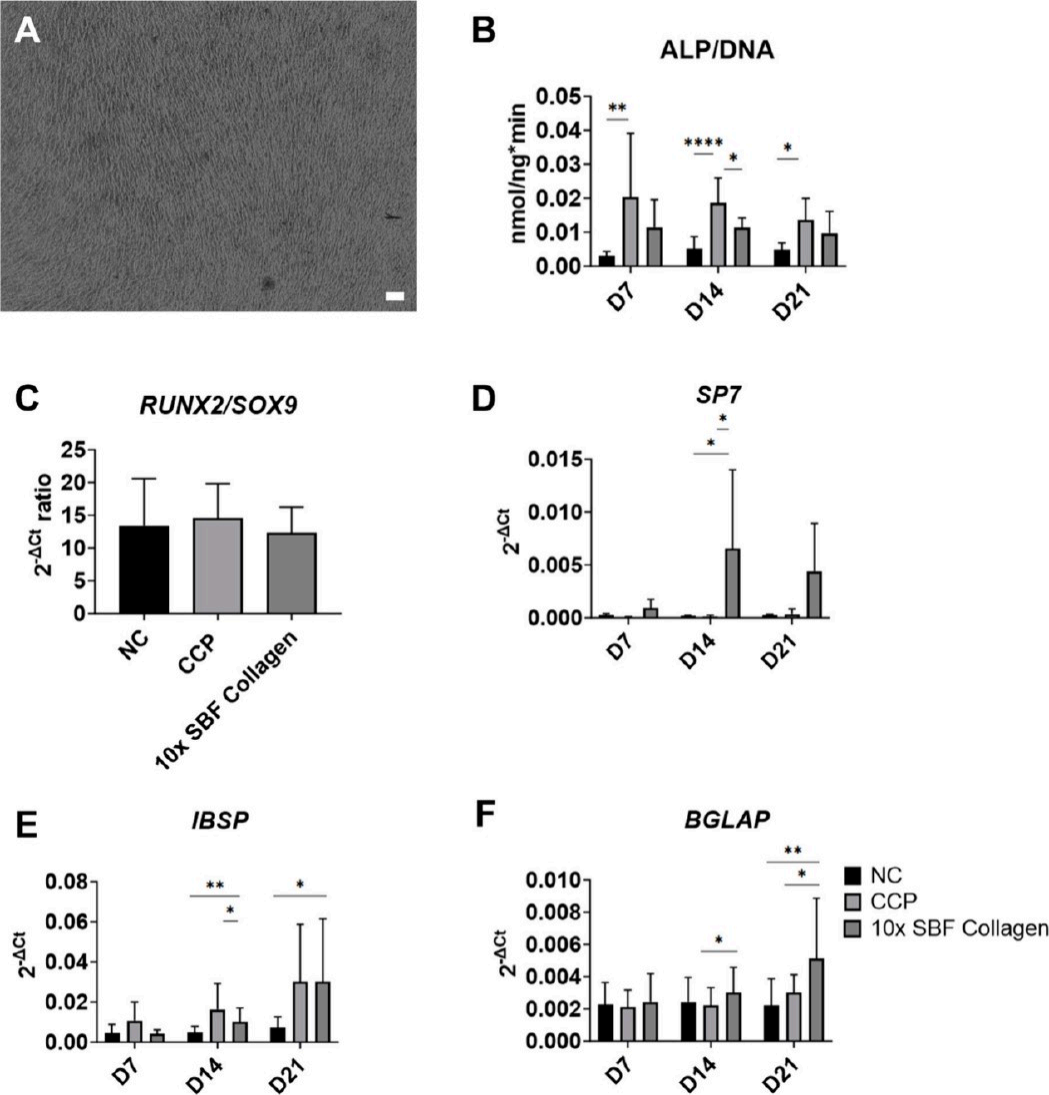
Osteogenic differentiation of BM-hMSCs (*n* = 3 donors) on 10× SBF collagen-coated CCP. (A) Phase contrast image of BM-hMSCs cultured under osteogenic conditions on 10× SBF collagen-coated CCP. Scale bar 100 μm. (B) ALP activity assay after 7 days, 14 days, and 21 days. (C) qPCR analysis of the *RUNX2*/*SOX9* expression ratio after 7 days. (D–F) qPCR analysis of *SP7* (D), *IBSP* (E), and *BGLAP* (F) expression at 7 days, 14 days, and 21 days. Statistical test: one way ANOVA with multiple comparison Bonferroni post-hoc test. Statistical significance: *p < 0.05, **p < 0.01, ****p < 0.0001.

Figure 7A shows a phase contrast image of BM-hMSCs cultured on a 10× SBF collagen coating after 21 days of differentiation, demonstrating the attachment of cells to the coated surface and their growth into a confluent monolayer. ALP activity was significantly lower in the 10× SBF collagen group compared to the CCP group on day 14 (Figure 7B). However, on days 7 and 21, no significant differences were observed, indicating similar ALP activity levels between the two groups at these time points.

At the gene expression levels, no significant differences in the *RUNX2*/*SOX9* ratio, an indicator of early osteogenic differentiation,^30,31^ were observed between growth substrates (Figure 7C). Significantly higher expression of *SP7* was detected in the 10× SBF collagen group compared to the CCP group on day 14 (Figure 7D). The gene expression levels of *IBSP* and *BGLAP* were similar between the CCP and 10× SBF collagen groups at all time points (Figure 7E and F).

### 3.5 10× SBF Collagen Coating Allows Real-Time Quantification of Mineral Formation and Resorption

**Figure 8:**
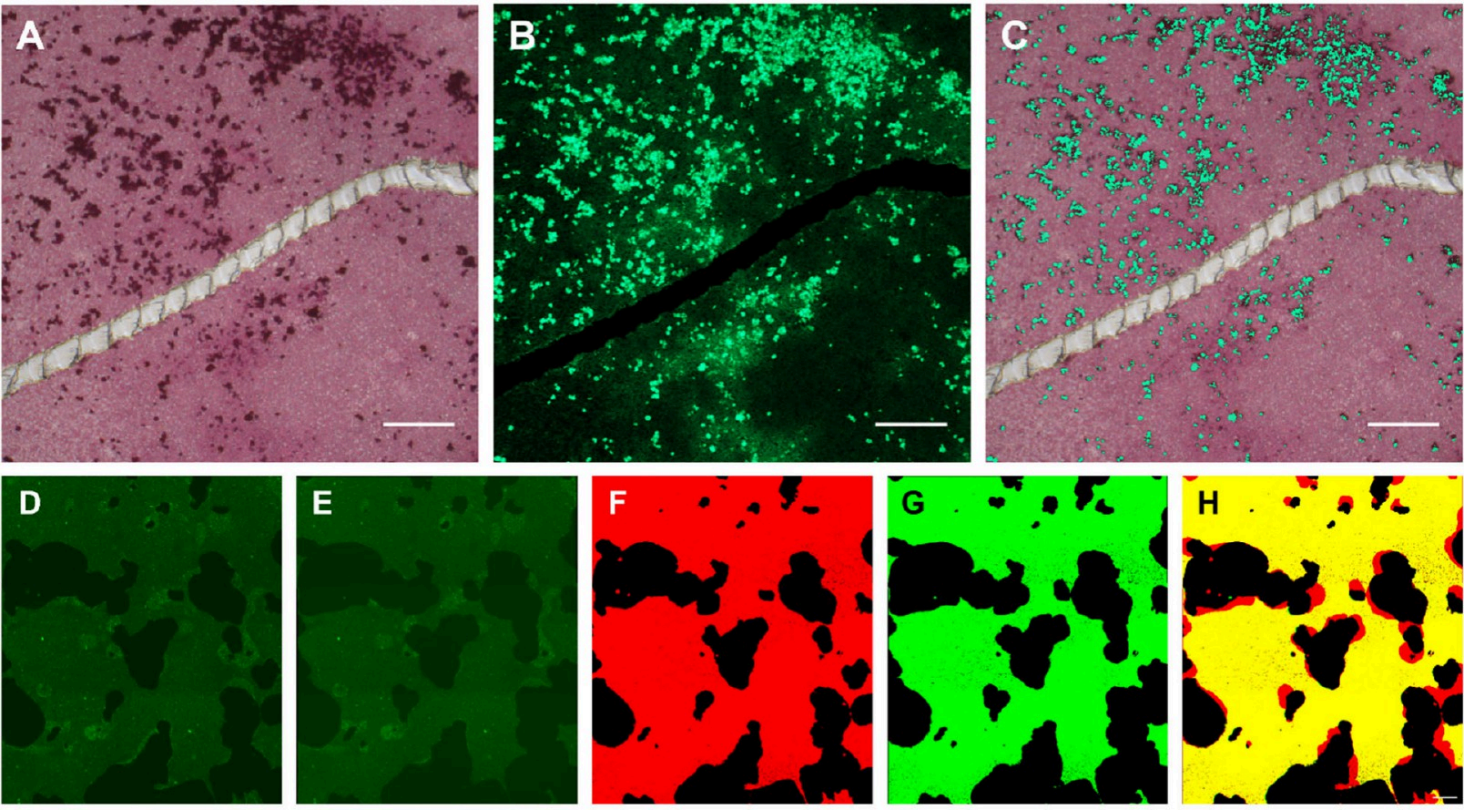
Assay development: Calcein Green and Alizarin Red staining of a coated surface after osteogenic culture of SaOS-2 cells for 4 weeks, *n* = 3. (A) Calcein Green staining. (B) Alizarin Red staining. (C) Overlay images of Calcein Green and Alizarin Red staining. Scale bar = 200 μm. (D) Osteoclasts seeded on a Calcein Green stained coating at the start of the time lapse recording. (E) Calcein Green stained coating after 22 h of time-lapse recording. (F) Thresholded image at the start of the time-lapse recording (coating in red, resorption sites in black). (G) Thresholded image at the end of the time-lapse recording (coating in green, resorption sites represented in black). (H) Merged and thresholded start and end images (remaining coating yellow and resorption sites in red).

The evaluation of osteoblast and osteoclast remodeling activity on 10× SBF collagen-coated substrates was performed using the fluorescent dye Calcein Green. The osteoblast-like SaOS-2 cell line was cultured on 10× SBF collagen-coated CCP for 4 weeks under osteogenic conditions. The purpose of this experiment was to demonstrate that newly formed minerals on the 10× SBF collagen coating could be visualized using Calcein Green staining (Figure 8). Alizarin Red staining, as the gold standard technique to detect mineralization *in vitro*, was then carried out to confirm this hypothesis. The Calcein staining in Figure 8A reveals a background with medium-intensity green fluorescence (the 10× SBF collagen coating), accompanied by bright fluorescent spots, which in turn can be attributed to cellular mineral deposits.

A mock resorption site was intentionally created by introducing a scratch on the surface of the coated substrate. This artificial resorption site was visually identifiable as a dark area located precisely at the site of the introduced scratch. The Alizarin Red staining in Figure 8B shows the coating in a medium red color, while the scratched area remained unstained. Cellular mineral deposits were intensively stained in red. Thus, both methods followed the same staining pattern, as confirmed by the overlay image in Figure 8C. This indicates that Calcein Green can be utilized to simultaneously visualize the formation and resorption of minerals on a 10× SBF coated substrate.

As osteoclast-mediated mineral resorption is a rapid process it was possible to monitor osteoclast activity in real time on 10× SBF collagen-coated substrates using Calcein Green (Figure 8D–H). Figure 8D shows the coated CCP substrate seeded with osteoclasts. The cells formed the first resorption sites at the start of the time-lapse recording, as indicated by the dark areas in the fluorescent coating. Figure 8E shows the same region of interest after 22 h of the time-lapse recording. To visualize the resorptive activity, the image at the start of the recording was thresholded, and the coating was pseudocolored in red (Figure 8F). The same process was applied to the end image of the recording with a green pseudocolor for the remaining coating (Figure 8G). The overlay of these images colored the remaining coating yellow, and the resorbed coating area red (Figure 8H). Time-lapse recording of osteoclasts allowed analysis of the dynamics of osteoclast-mediated activity and collection of cell-specific data sets such as “Resorptive activity per hour″ or “Resorptive activity per osteoclast” (Table 3). A video of the time-lapse recording is provided in the Supporting Information (Videos S1 and S2). This provides proof of concept level confirmation of our ability to assess resorptive activity via image analysis.

**Table 3:** Resorptive Activity of Osteoclasts within 22 h of Culture^a^

Area 0 h [μm^2^]	Area 22 h [μm^2^]	Resorbed area [μm^2^]	Resorptive activity per hour [μm^2^/h]	Resorptive activity per osteoclast [μm^2^/h/OC]
537157	462546	74611	3391	81

a n = 1.

### 3.6 Quantification of Mineral Formation and Resorption in an Osteoblast–Osteoclast Coculture Model

**Figure 9:**
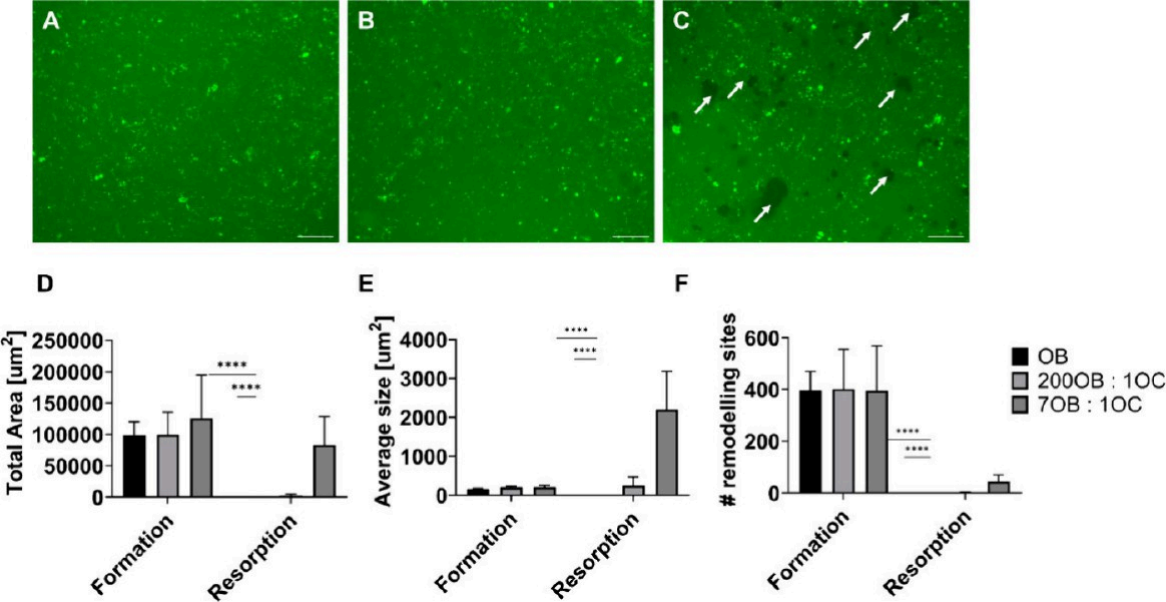
Osteoblast (OB) and osteoclast (OC) coculture with the predifferentiation step after 7 days of coculture. Cells were cultured on the 10× SBF collagen substrates and samples were stained with Calcein Green to evidence mineral deposition and resorption. (A) Osteoblast monoculture. (B) OB:OC ratio 200:1. (C) OB:OC ratio 7:1. The resorption sites are indicated by white arrows. (D) Quantification of the total remodeled area. (E) Quantification of the number of remodeling sites. (F) Quantification of the average remodeling site sizes. The scale bar is 100 μm. Statistical test: one way ANOVA with multiple comparison Bonferroni post-hoc test. OB, *n* = 9. OB:OC ratio 200:1, *n* = 8. OB:OC ratio 7:1, *n* = 12. Statistical significance: ****p < 0.0001.

Cocultures were generated by seeding BM-hMSC-derived osteoblasts and BM-MNCs derived osteoclasts on 10× SBF collagen-coated CCP. The investigated ratios were OB:OC 200:1, OB:OC 7:1, and an osteoblast monoculture. The representative images in Figure 9 show the calcein stained 10× SBF collagen-coated CCP surfaces after 7 days of coculture. All three culture systems showed abundant bright fluorescent spots, indicating cellular mineral formation (Figure 9A, B, and C). The osteoblast monoculture and 200:1 coculture showed none or few signals for mineral resorption (Figure 9A,B). Significant resorptive activity was only detected in the 7:1 coculture (Figure 9C). Images were then thresholded and quantified for mineral formation and resorption (Figure 9D,E,F). No significant differences between the groups concerning mineral formation in terms of “remodeling area” (Figure 9D), “number of remodeling sites” (Figure 9 E) and “average size of remodeling sites” were detected (Figure 9F). Quantification of mineral resorption showed that the assessed resorption parameters increased with increasing osteoclast number in the coculture (Figure 9 D, E, F).

A 2D osteoblast-osteoclast coculture model was established using the described methodology, in which cellular mineral formation and resorption were quantified within the same culture well on 10× SBF collagen-coated CCP. The OB:OC 7:1 ratio was the most suitable to follow the remodeling activity.

## 4 Discussion

### 4.1 Cellular Performance on 10× SBF Collagen Coating

The 10× SBF collagen coating technique provides a biomimetic bone-like microenvironment on CCP, consisting of carbonated apatitic calcium phosphate particles and collagen type I. The combination of SEM, XRD, EDX, and ATR-FTIR analyses provided evidence for the formation of a composite coating composed of amorphous carbonate-substituted apatitic calcium phosphate-and type I collagen on the material substrate, utilizing the 10× SBF collagen coating formulation. Carbonates can be introduced at two anionic sites in the apatitic calcium phosphate crystal structure. Substitution at the hydroxyl site is called A-type, whereas substitution at the phosphate site is classified as B-type carbonated apatitic calcium phosphate.^32^ This indicates a promoted formation of B-type carbonated apatitic calcium phosphate as the B-type substitution results in a lower phosphate content, which leads to a higher Ca/P ratio.^33^ Comparing this to the 10× SBF coating, it indicates that the inclusion of collagen resulted in a greater degree of substitution, leading to an increased Ca/P ratio and a more pronounced amorphous nature of the deposited coating.

A major limitation of existing calcium phosphate-coated surfaces is their inadequate support for cell attachment and proliferation, which are prerequisites for the successful osteogenic differentiation of hMSCs.^34−36^ Compared to other calcium phosphate-coated surfaces (10× SBF and Corning Osteo Assay Surface), the 10× SBF collagen coating overcomes this challenge by significantly enhancing hMSC attachment and proliferation, and thus represents a superior growth substrate for bone cell culture applications. The observed superior cell adhesion properties of this composite coating are attributed to the presence of collagen, which provides bioactive motifs such as GFOGER, known to promote integrin-mediated cell attachment.^37^ This enhanced attachment directly correlates with increased proliferation and reduced cell death, as evidenced by lower LDH release and flattened cell morphology indicative of a strong substrate interaction.^38^ There have been both positive and negative reports on the effects of plain calcium phosphate-coated substrates on the growth of preosteoblastic cell types. Similar to our findings, Nandakumar et al. showed that proliferation of primary hMSCs was higher on CCP than on calcium phosphate-coated (5x SBF) PCL substrates during 14 days of culture.^35^ In addition, another report suggested a negative effect of calcium phosphate growth substrates on cell viability and proliferation. The authors cultured mouse MC3T3-E1 preosteoblasts on CCP and three different calcium phosphate coating types and showed that CCP promoted the greatest proliferation over a 14-day culture period.^34^ In contrast to the aforementioned results, Iijima et al. and Andric et al. described similar cell growth of hMSCs on CCP-and calcium phosphate-coated surfaces.^39,40^ However, in the study by Andric et al., metabolic activity, as a measure of cell number, was not normalized to the available cell growth area, and hence offers a limited comparison of the 2D CCP control and the 3D calcium phosphate-coated substrate.^40^

In addition to enhanced cellular adhesion and proliferation, the 10× SBF collagen coating also supported robust osteogenic differentiation of BM-hMSCs. Unlike the Corning Osteo Assay Surface, which led to detachment and spheroid formation of differentiating cells, the 10× SBF collagen coating maintained a stable monolayer culture throughout the differentiation process. The adhesion motifs present in the coating likely contributed to this enhanced substrate affinity. The varying substrate affinities and the resulting spheroid or monolayer formation complicate the comparison of both mineral substrates’ effects on osteogenic differentiation. This is because spheroid culture itself has been shown to enhance the osteogenic potential of MSCs compared to monolayer culture.^41^ We assume that adhesion motifs (such as GFOGER) within the 10× SBF collagen coating increased the cell affinity to the substrate in comparison to the Osteo Assay Surface, which is devoid of such motifs. Thus, the 10× SBF collagen coating allowed for long-term culture and osteogenic differentiation in a monolayer. Our findings align with previous studies demonstrating that mineralized collagen scaffolds provide essential cues for osteogenic differentiation by supporting increased expression of osteogenic marker and promoting matrix mineralization.^42^ In this study, the authors observed enhanced osteogenic differentiation on collagen and mineralized collagen membranes, as evidenced by increased mRNA expression levels of *SPP1*, *RUNX2*, *ENPP1*, and *BGLAP* after 3 days of culture. Similarly, Mazzoni et al. demonstrated that a hydroxyapatite collagen composite scaffold (Coll/Pro Osteon 200) supported the osteogenic differentiation of hMSCs, as shown by increased matrix mineralization and osteocalcin expression, even without the addition of osteogenic factors to the culture medium.^43^ Taken together, these studies highlight the ability of apatitic calcium phosphate collagen composite materials to support the osteogenic differentiation of hMSCs.

Beyond osteogenic differentiation, the 10× SBF collagen coating also plays a crucial role in osteoclast differentiation and activity assessment. The differentiation of osteoclasts from BM-MNCs was successfully achieved on the composite coating, and, importantly, resorption activity was observed and quantified both as end point or in real time using the fluorophore Calcein Green. Since osteoclast differentiation is highly donor-dependent, the ability to monitor and quantify resorption activity during cell culture provides valuable insights into the degree of osteoclast maturation. This presents a significant advantage over other mineralized collagen substrates, as it enables the standardization of osteoclast differentiation based on a defined degree of resorption. This is particularly important for drug screening, where osteoclast resorption activity should exceed 10% of the resorbed area.^44^

Our findings are consistent with a previous report by Ciapetti et al., who demonstrated that the differentiation of osteoclasts from peripheral blood mononuclear cells was impeded when cells were seeded on hydroxyapatite-coated surfaces, and that CCP was comparably more effective for differentiation.^45^ In agreement with these results, gene expression of osteoclast marker genes *Ctsk*, *Ca2* and *Mmp9* was reduced when murine RAW 264.7 macrophages were seeded on β-tricalcium phosphate and calcium-deficient hydroxyapatite-coated surfaces.^46^ Melo Pereira et al. demonstrated that osteoclasts differentiated on biomineralized collagen membranes were unable to resorb the mineralized substrates.^27^ The authors postulated that the absence of RGD motifs in the biomineralized collagen membranes may lead to the formation of nonresorbing osteoclasts. In contrast, our study observed the resorptive activity of osteoclasts differentiated on a collagen-apatitic calcium phosphate composite coating. Hence, we assume that rather the porous mesh-like surface topography of the biomineralized collagen membranes used by Melo Pereira et al. induced the formation of nonactive osteoclasts rather than the biochemical aspects of the surface. However, additional investigations are needed to determine whether variations in surface topology, disparities in the calcium phosphate phase composition, or differences in collagen content may have led to the disparate outcomes observed between studies.

The bone cell environment within the human body is highly complex and dynamic, involving intricate interactions with various cell types, extracellular matrix components, and biochemical signals. In summary, we successfully developed a coating methodology that generated a bone-like microenvironment on CCP. The osteogenic differentiation of BM-hMSCs and osteoclastic differentiation of BM-MNCs observed on mineral coatings appear to be more physiological than that on CCP. This difference is attributed to the presence of essential microenvironmental cues, namely, type I collagen and apatitic calcium phosphate, which contribute to the generation of a bone-like extracellular environment. The absence of such crucial physiological cues on CCP may lead to different cell differentiation patterns compared with a more bone-like microenvironment.

### 4.2 Coculture Model and Cellular Remodeling Quantification

Using the fluorescent dye Calcein Green, we monitored the activity of osteoblasts and osteoclasts in real time in mono- and cocultures on 10× SBF collagen-coated CCP. To our knowledge, this is one of the few *in vitro* osteoblast-osteoclast coculture models that allows simultaneous real-time monitoring of cellular mineral formation and resorption on a biomimetic substrate.^47^

Alizarin Red and Von Kossa staining are gold standard techniques for the detection and quantification of mineral deposition *in vitro*. However, these two methods can only be utilized as end point measurement techniques as they require sample fixation and are not suitable for high-throughput applications owing to their time-consuming quantification. The use of Calcein dyes for visualizing and studying bone remodeling has been extensively documented by various research groups. Rahn and Perren conducted a study in which Calcein Blue was injected into animals and incorporated into the remodeled bone, offering a means to study bone remodeling *in vivo*.^18^ Serguienko et al. utilized Calcein Green to monitor and quantify mineralization during the osteogenic differentiation of human hMSCs *in vitro*.^19^ Their findings align with the results presented herein, as both studies demonstrated the comparable sensitivity of Alizarin Red and Calcein Green in detecting mineral deposits.

Similarly, Wang et al. observed a correlation between fluorescent mineral labeling using Calcein and von Kossa staining during the culture of mouse osteoblasts.^20^ However, both Serguienko and Wang’s studies primarily focused on quantifying mineral formation. Our results extend upon these findings by demonstrating that not only mineral formation, but also mineral resorption can be quantified simultaneously in monocultures of osteoblasts or osteoclasts, as well as in coculture systems. Other studies reported the use of X-ray computed microtomography to quantify the bone matrix turnover of osteoblasts and osteoclasts cultured on 3D scaffolds.^22,48,49^ However, this method is time-consuming, cannot be performed in a 2D multiwell format, and is thus not appropriate for high-throughput applications.

In the native bone microenvironment, osteoclasts and osteoblasts interact not only with each other but with various other cell types within the basic multicellular unit (BMU) to regulate bone homeostasis including osteocytes, bone lining cells, OsteoMacs, and vascular endothelial cells.^50^ While our study focused on the direct effects of the 10× SBF collagen coating on osteoblasts and osteoclasts, it is conceivable that the developed biomimetic composite coating could also influence other bone-resident cell types *in vitro*. Osteocytes, for example, are mechanosensitive cells embedded in the mineralized matrix that play a crucial role in regulating bone remodeling through RANKL and sclerostin signaling. The ability of our coating to support osteoblast and osteoclast function suggests that it may also provide an appropriate microenvironment for osteocytes to integrate into long-term cultures. Similarly, bone lining cells and OsteoMacs, which contribute to osteoblast recruitment and bone turnover, may respond to the surface properties of the coating, potentially enhancing their role in bone remodeling. Additionally, vascular endothelial cells are essential for coupling angiogenesis with osteogenesis, and future studies could investigate whether our biomimetic coating can support endothelial cell adhesion and function, thereby enabling the development of a more physiologically relevant bone model. Exploring these interactions in future studies, for example with dynamic on chip culture systems, could provide further insights into the full potential of the 10× SBF collagen coating in replicating the complexity of the bone microenvironment.

Although our coculture model uses a bone-like cell culture substrate, it lacks a 3D extracellular environment and thus does not fully mimic the complex 3D environment of native bone tissue. Other groups have reported the generation of more sophisticated three-dimensional (3D) bone models that combine osteoblasts and osteoclasts. However, in terms of use of the technique as a practical assay this is a trade-off rather than a limitation, as quantifying mineral formation and resorption in a 3D environment remains difficult, especially at the throughput rates required for the evaluation of candidate compounds in drug development.

### 4.3 Conclusion

Our study demonstrates that the 10× SBF collagen coating significantly enhanced cellular attachment, viability, proliferation, and osteogenic differentiation compared to plain hydroxyapatite-coated substrates. Unlike the 10× SBF and Corning Osteo Assay Surface, which exhibited limitations in supporting stable osteogenic differentiation due to cell detachment, the 10× SBF collagen coating provided a robust substrate for long-term culture. Additionally, in contrast to CCP, which remains the most effective for osteoclast generation, the 10× SBF collagen coating offers the advantage of supporting osteoclast differentiation in a more physiologically relevant environment while also enabling the visualization and quantification of their resorptive activity. This feature is particularly important given the donor-dependent variability in osteoclast differentiation.

Moreover, our findings suggest that the 10× SBF collagen coating, in combination with Calcein Green and osteoblast-osteoclast coculture, provides a novel approach for investigating human osteoblast-osteoclast remodeling activity in a 2D setting. This model permits the simultaneous quantification of bone formation and resorption, offering a valuable tool for studying dynamic bone cell interactions. In the future, we plan to validate the coculture model for drug screening applications using established osteoporosis medications such as the resorption inhibitor calcitonin or zoledronic acid. This approach has the potential to be exploited in high-throughput drug discovery applications, enabling the development of novel osteoporosis therapeutics and diagnostics.

## Supporting Information Available

Video S1 showing time lapse imaging of osteoclasts on 10× SBF collagen coating for 22 h, phase contrast (MP4)
Video S2 showing time lapse imaging of osteoclasts on 10× SBF collagen coating for 22 h, calcein green staining (MP4)
Figure S2 showing cross-sectional SEM image of 10× SBF collagen coating on CCP after 120 min (PDF)
