# Supplementary material for: Developing a Bone-Mimicking Microenvironment: Surface Coating Method for Investigating Bone Remodeling in Vitro
Source: ACS Biomater Sci Eng. 2025 Apr 10;11(5):2690–704. doi: 10.1021/acsbiomaterials.4c02330 (PMC12076279; doi:10.1021/acsbiomaterials.4c02330)
Supplement: Supplementary file 3 — ab4c02330_si_003.pdf [file ab4c02330_si_003.pdf]

Developing a Bone-Mimicking Microenvironment: Surface Coating Method for Investigating Bone Remodeling in Vitro.

A. Sieberath,[a,c,d,*] D. Eglin,[b] CM. Sprecher,[c] AM. Ferreira,[a] P. Gentile,[a] K. Dalgarno,[a] E. Della Bella[c]

[a] Newcastle University, Newcastle upon Tyne NE1 7RU, UK; [b] Mines Saint-Étienne, INSERM, U1059 Sainbiose, Saint-Étienne, FR; [c] AO Research Institute,7270 Davos, CH; [d] University Hospital Knappschaftskrankenhaus Bochum, 44892 Bochum, DE

*Email: alexander.sieberath@rub.de

Time lapse imaging of Osteoclasts on 10x SBF collagen coating

Video S1 (Calcein green staining).mp4   Video S1 (phase contrast).mp4

Video S1: Time lapse imaging of osteoclasts on 10x SBF collagen coating for 22h. (A) Timelapse imaging of calcein stained 10x SBF collagen coating. (B) Phase contrast time lapse imaging. Scale bar 100 µm

Coating thickness analysis

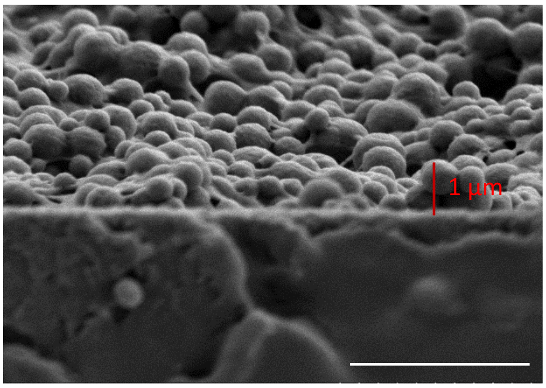

Figure S2: Cross-sectional SEM image of 10x SBF collagen coating on TCP after 120 min. Scale bar 3 µm.
